# Supplementary material for: Structures of TGF-β with betaglycan and signaling receptors reveal mechanisms of complex assembly and signaling
Source: Nat Commun. 2025 Feb 26;16:1778. doi: 10.1038/s41467-025-56796-9 (PMC11865472; doi:10.1038/s41467-025-56796-9)
Supplement: Supplementary file 1 — Supplmentary Information [file 41467_2025_56796_MOESM1_ESM.pdf]

Supplementary Information

Structures of TGF-β with betaglycan and signaling receptors reveal mechanisms of complex assembly and signaling

Łukasz Wieteska[1,2,3], Alexander B. Taylor[4], Emma Punch[5], Jonathan A. Coleman[1], Isabella O. Conway[1], Yeu-Farn Lin[6], Chang-Hyeock Byeon[1], Cynthia S. Hinck[1], Troy Krzysiak[1], Rieko Ishima[1], Fernando López-Casillas[7], Peter Cherepanov[5], Daniel J. Bernard[6], Caroline S. Hill[3,*] and Andrew P. Hinck[1,*]

[1]Department of Structural Biology, University of Pittsburgh, Pittsburgh, PA, USA

[2]Faculty of Biological Sciences, Astbury Centre for Structural Studies, University of Leeds, Leeds, UK

[3]Developmental Signalling Laboratory, The Francis Crick Institute, London NW1 1AT, UK

[4]Department of Biochemistry & Structural Biology and Greehey Children's Cancer Research Institute, The University of Texas Health Science Center at San Antonio, TX, USA

[5]Chromatin Structure and Mobile DNA Laboratory, The Francis Crick Institute, London NW1 1AT, UK

[6]Department of Pharmacology and Therapeutics, McGill University, Montreal, QC, Canada

[7]Departmento de Biología Celular y del Desarrollo, Instituto de Fisiología Celular, UNAM, Mexico City, Mexico

[*]Corresponding Authors:

Prof. Andrew P. Hinck, Department of Structural Biology, University of Pittsburgh School of Medicine, Biomedical Science Tower 3, Room 2051, 3501 Fifth Avenue, Pittsburgh, PA 15260, U.S.A, Telephone: +1(412) 648-8533, E-mail: ahinck@pitt.edu

Caroline S. Hill, Developmental Signalling Laboratory, The Francis Crick Institute, London NW1 1AT, UK, Telephone: +44 (0)20 3796 1251, E-mail: caroline.hill@crick.ac.uk

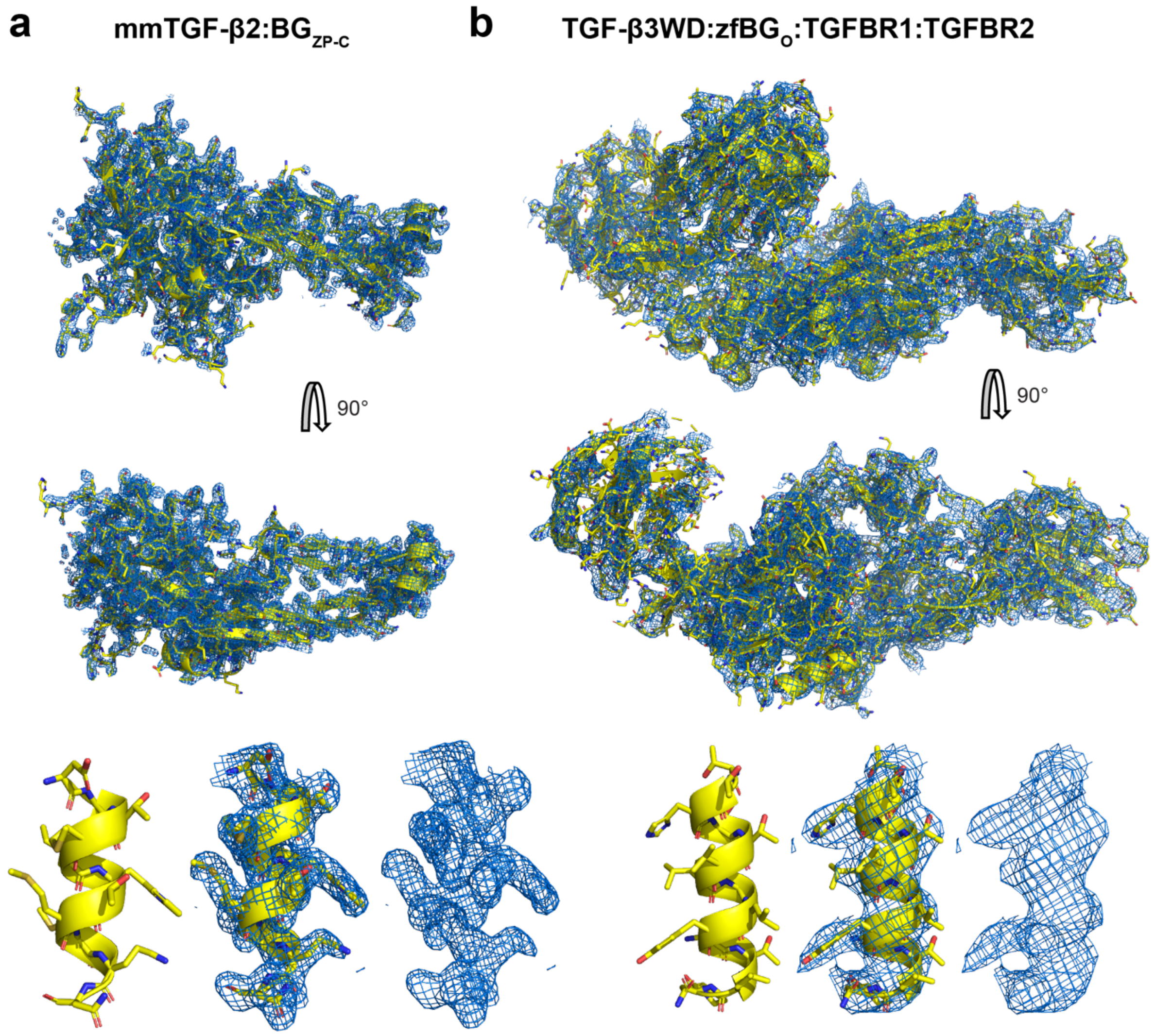

Supplementary Figure 1. Electron density maps of the presented structures.

(**a**, **b**) 2Fo-Fc electron density maps for mmTGF-β2:BG$_{ZP-C}$ (**a**) and TGF-β3WD:zfBG$_{O}$:TGFBR1:TGFBR2 (**b**) contoured at 1.0 σ, are displayed as blue mesh representations with a carve distance of 2 Å around the atomic model. Zoomed-in views highlight the quality of the electron density surrounding the FG-helix (**a**) or the heel helix (**b**).

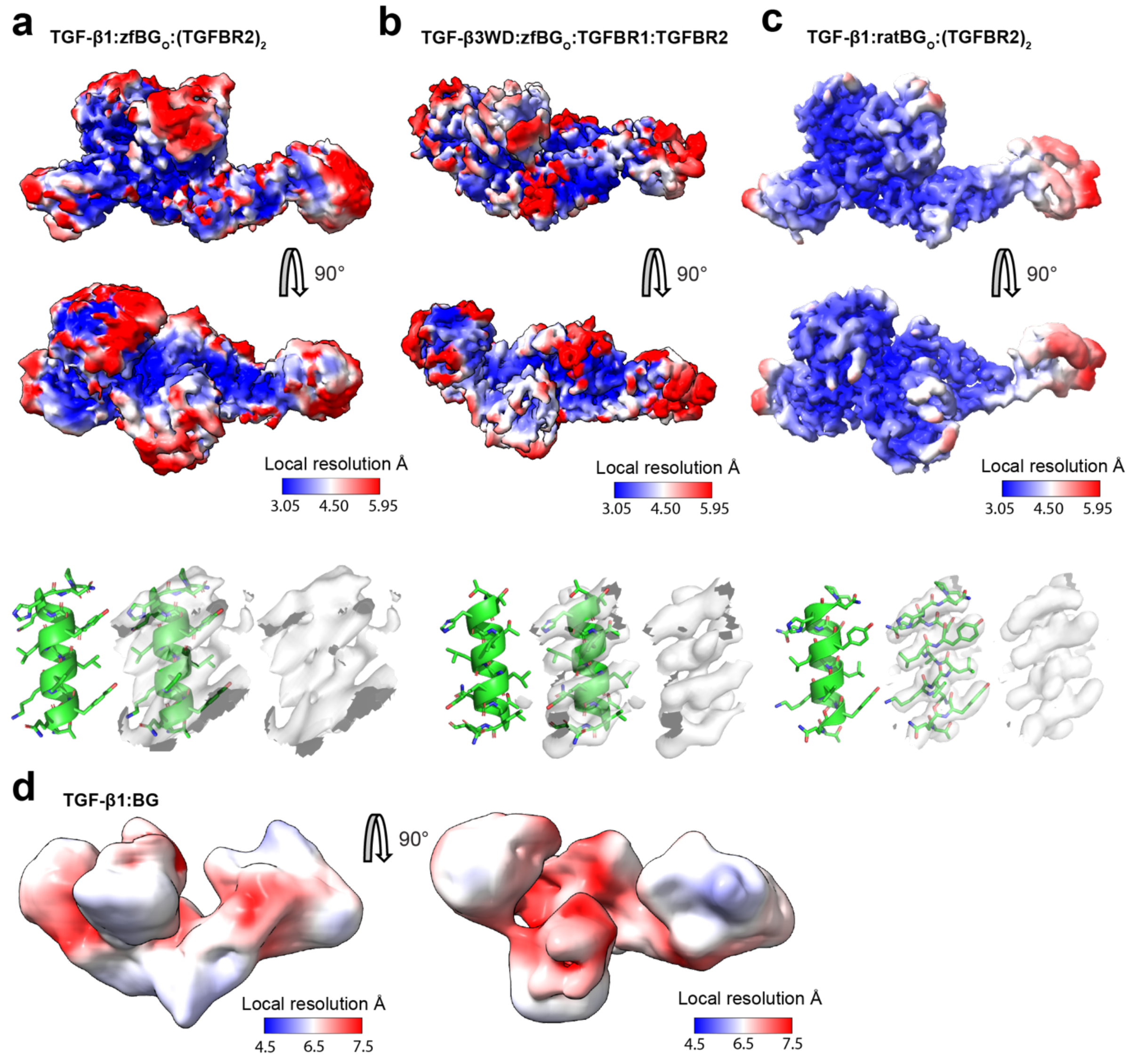

Supplementary Figure 2. Cryo-EM maps colored by local resolution.

(**a** - **d**) Cryo-EM maps colored by local resolution for the following complexes: TGF-β1:zfBG$_O$:(TGFBR2)$_2$ (**a**), TGF-β3WD:zfBG$_O$:TGFBR1:TGFBR2 (**b**), TGF-β1:ratBG$_O$:(TGFBR2)$_2$ (**c**) and TGF-β1:BG (**d**). For (**a** - **c**), corresponding zoomed-in panels highlight the density of the heel helix of the TGF-β monomer adjacent to that bound to BG$_O$.

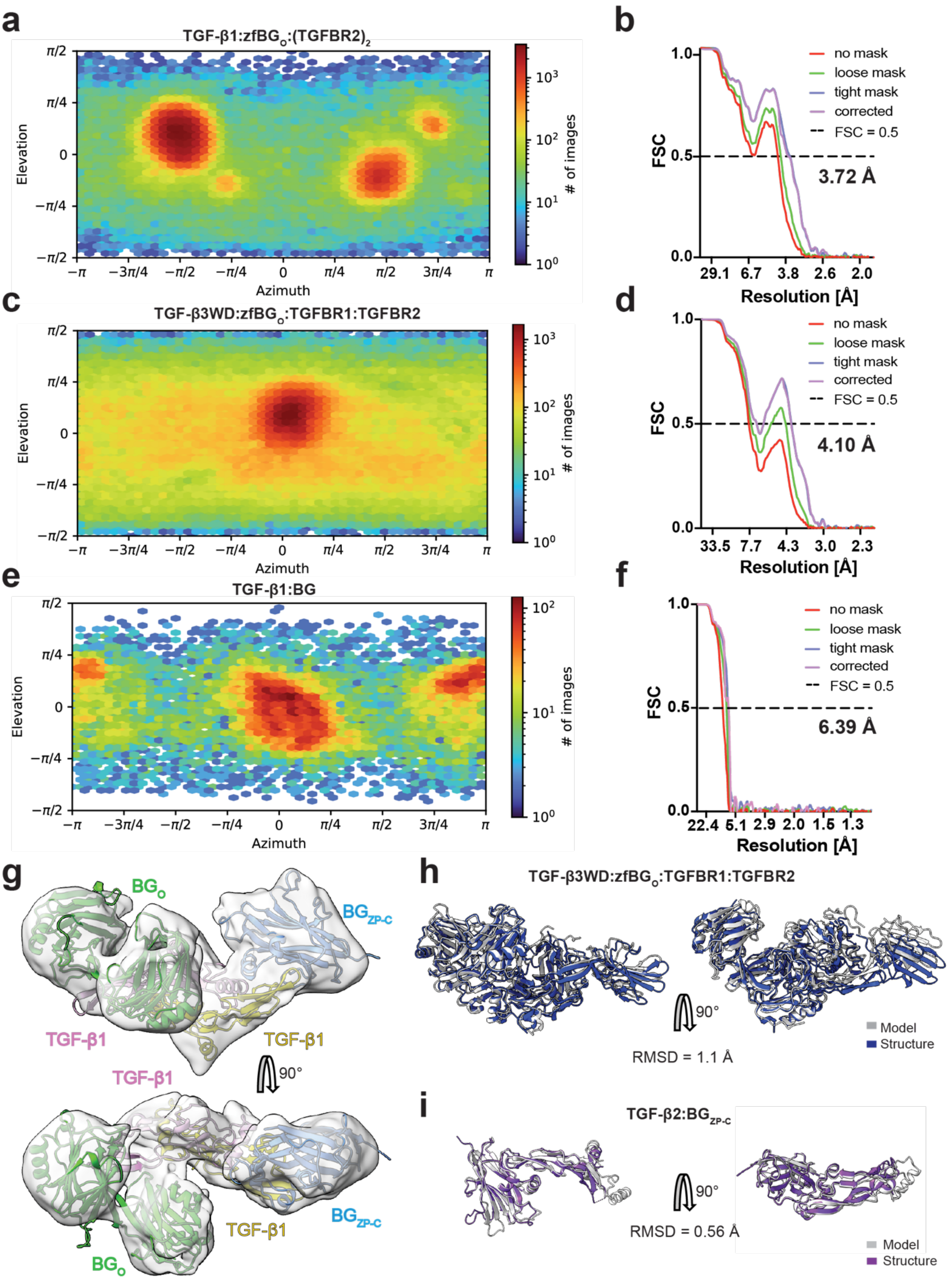
a
TGF-β1:zfBGo:(TGFBR2)2
Elevation
Azimuth
# of images
b
FSC
Resolution [Å]
no mask
loose mask
tight mask
corrected
FSC = 0.5
3.72 Å
c
TGF-β3WD:zfBGo:TGFBR1:TGFBR2
d
4.10 Å
e
TGF-β1:BG
f
6.39 Å
g
BGo
BGZP-C
TGF-β1
90°
h
TGF-β3WD:zfBGo:TGFBR1:TGFBR2
RMSD = 1.1 Å
Model
Structure
i
TGF-β2:BGZP-C
RMSD = 0.56 Å

Supplementary Figure 3. Structural details of the Cryo-EM complexes and AF2M models.

(**a** - **f**) Particle view distributions for presented 3D reconstructions of TGF-β1:zfBG$_O$:(TGFBR2)$_2$ (**a**), TGF-β3WD:zfBG$_O$**:**TGFBR1:TGFBR2 (**c**), or TGF-β1:BG (**e**), together with Fourier Shell Correlation (FSC) curves (**b**, **d**, **f**, respectively).

(g) Low resolution Cryo-EM map of the TGF-β:BG complex with fitted model to the density.

(**h**, **i**) AF2M models of TGF-β3WD:BG$_O$:TGFBR1:TGFBR2 (**h**) and TGF-β2:BG$_{ZP-C}$ (**i**). The complexes are overlaid with crystal structures of TGF-β3WD:zfBG$_O$:TGFBR1:TGFBR2 and mmTGF-β2:BG$_{ZP-C}$, respectively.

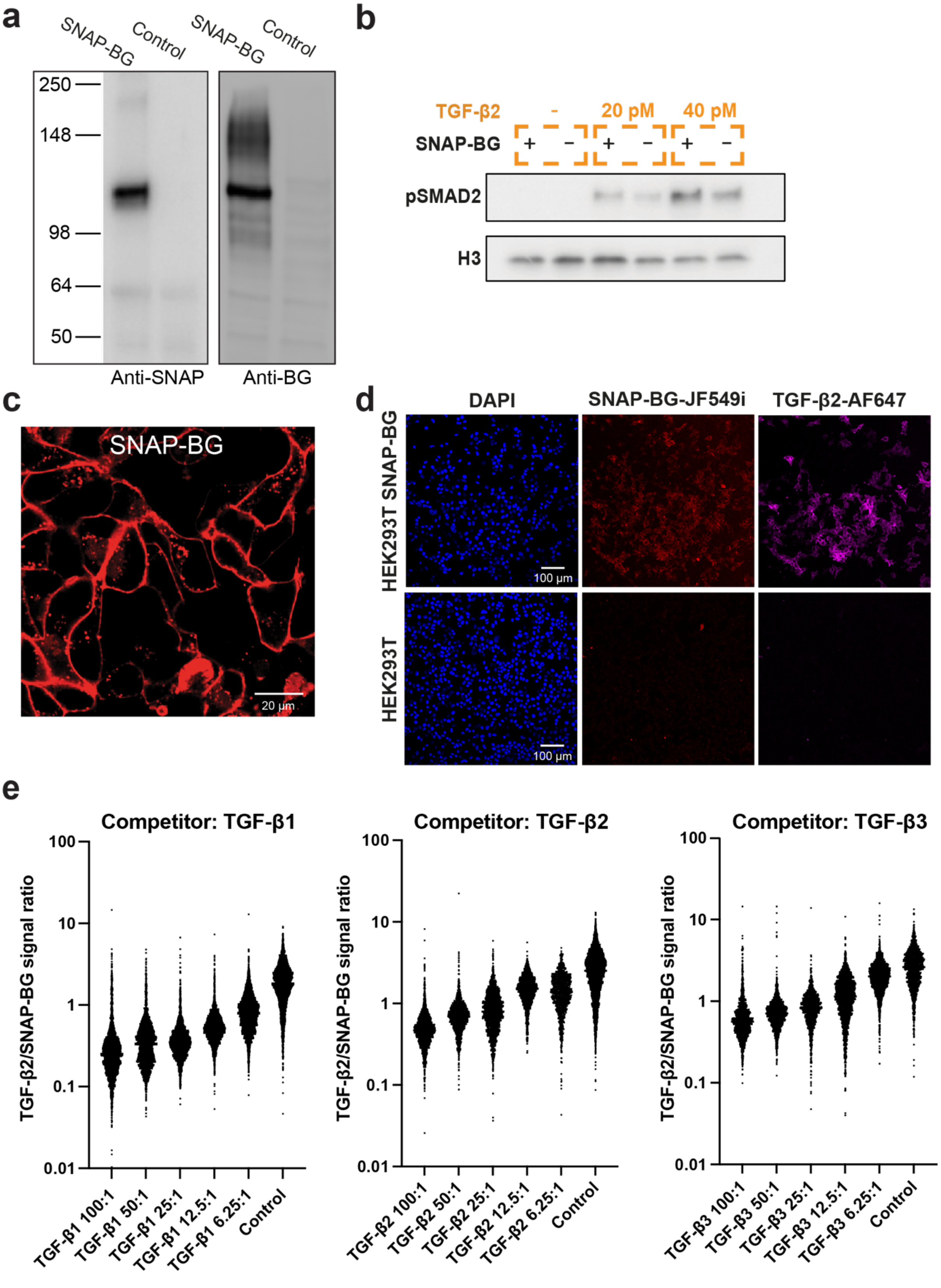

a
SNAP-BG
Control
SNAP-BG
Control
250
148
98
64
50
Anti-SNAP
Anti-BG
b
TGF-β2
-
20 pM
40 pM
SNAP-BG
+
−
+
−
+
−
pSMAD2
H3
c
SNAP-BG
20 µm
d
DAPI
SNAP-BG-JF549i
TGF-β2-AF647
HEK293T SNAP-BG
HEK293T
100 µm
100 µm
e
Competitor: TGF-β1
Competitor: TGF-β2
Competitor: TGF-β3
TGF-β2/SNAP-BG signal ratio
100
10
1
0.1
0.01
TGF-β1 100:1
TGF-β1 50:1
TGF-β1 25:1
TGF-β1 12.5:1
TGF-β1 6.25:1
Control
TGF-β2 100:1
TGF-β2 50:1
TGF-β2 25:1
TGF-β2 12.5:1
TGF-β2 6.25:1
Control
TGF-β3 100:1
TGF-β3 50:1
TGF-β3 25:1
TGF-β3 12.5:1
TGF-β3 6.25:1
Control

Supplementary Figure 4. Characterization of SNAP-betaglycan (SNAP-BG) in HEK293T cells.

(**a**) Western blot analysis of SNAP-BG expression in stably transfected HEK293T cells with pCDNA3.1 plasmid harbouring SNAP-BG. Proteins were resolved by SDS-PAGE and probed with an antibody specific to the SNAP-tag (left panel) or BG (right panel). A distinct band is observed at approximately 120 kDa, consistent with the expected molecular weight of the ΔGAG SNAP-BG fusion protein. Additionally, diffuse signal above 120 kDa likely reflects the presence of highly glycosylated population. The anti-BG antibody does not detect low levels of endogenous protein in these cells.

(**b**) Western blot analysis of L6E9 cell lysate demonstrates a dose-dependent increase in phosphorylated SMAD2 (pSMAD2) levels in the cells transfected with SNAP-BG upon stimulation with TGF-β2. Histone H3 acts as a loading control.

(**c**) SNAP-BG is membrane localized. Confocal microscopy images showing SNAP-BG conjugated with a fluorescent ligand (shown in red), predominantly localized on the cell membrane. Scale bar, 20 μm.

(**d**) Confocal microscopy images of HEK293T cells stably transfected with a SNAP-tagged BG construct (upper row) or untransfected control cells (bottom row) stained with DAPI to detect the nuclei (left panels, blue), SNAP-tag ligand conjugated to the JF549i fluorescent dye (middle panels, red), along with TGF-β2 tagged with AF647 fluorescent dye (right panels, violet). Control, untransfected cells present no signal either from SNAP-BG or TGF-β2. Scale bar, 100 μm.

(**e**) Concentration-dependent effects of TGF-βs as competitors in the binding assay. As the concentration of each TGF-β isoform increases (TGF-β1, TGF-β2, TGF-β3), there is a corresponding decrease in the colocalization signal ratio of TGF-β2-CF640R/SNAP-BG, indicating assay sensitivity to ligand concentration. Each data point represents the ratio of SNAP-BG signal to InhA signal within a segmented region of the image generated by applying the Otsu thresholding method to the SNAP-BG channel. The analysis was performed on three images per condition, each corresponding to an independent biological replicate (n=3).

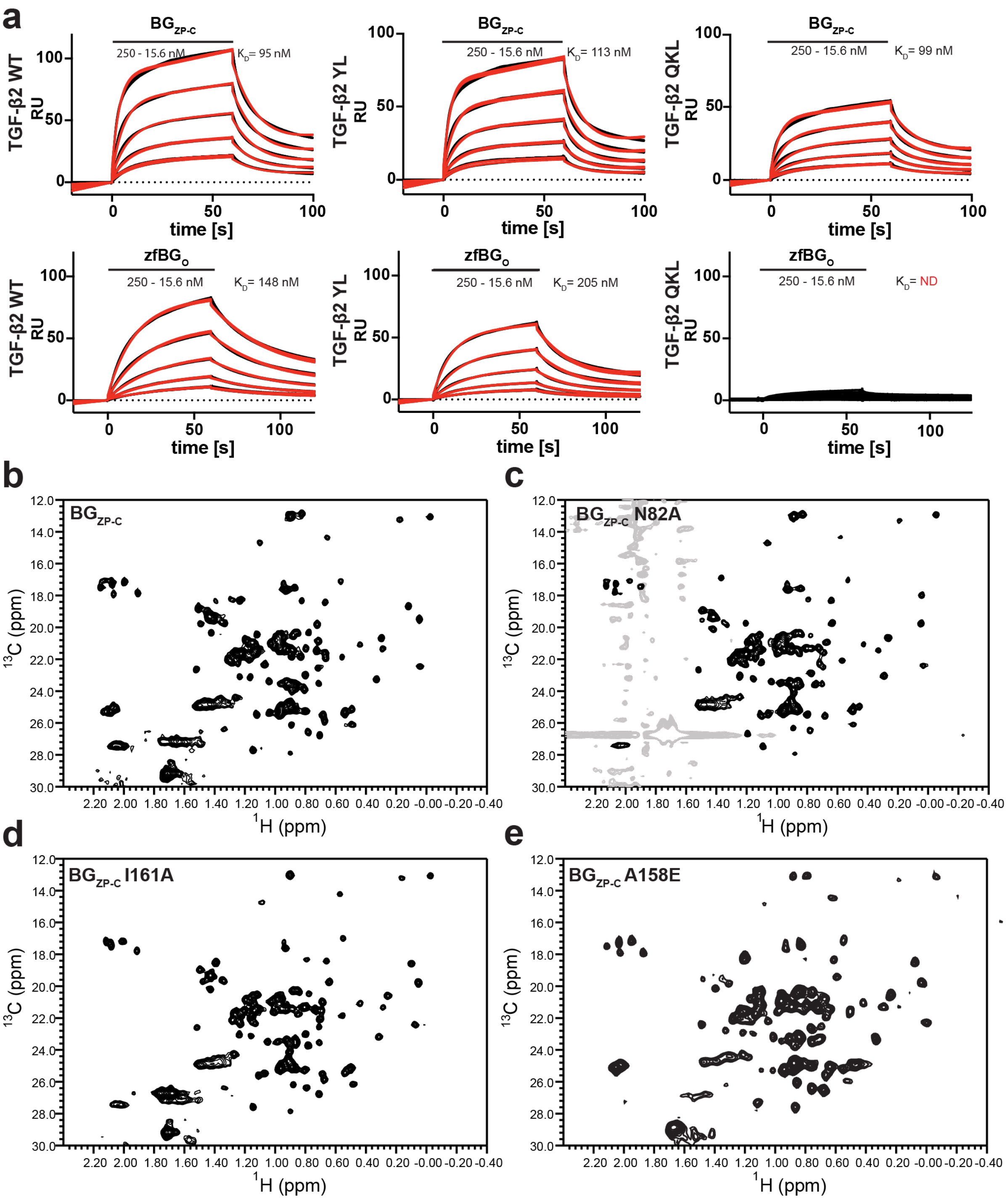
a
BGZP-C
250 - 15.6 nM
KD= 95 nM
KD= 113 nM
KD= 99 nM
TGF-β2 WT RU
TGF-β2 YL RU
TGF-β2 QKL RU
time [s]
zfBGO
KD= 148 nM
KD= 205 nM
KD= ND
b
BGZP-C
c
BGZP-C N82A
d
BGZP-C I161A
e
BGZP-C A158E
13C (ppm)
1H (ppm)

Supplementary Figure 5. Mutations affecting TGF-β2 binding cause only local structural changes.

(**a**) Sensorgrams from SPR experiment where zfBG$_O$ WT or a BG$_{ZP\text{-}C}$ WT 2-fold dilution series (black) were injected over immobilized TGF-β2 WT, TGF-β2 (Y50A, L51A located in pre-helix extension), or TGF-β2 (Q57A, K60A, L64A located on heel helix). zfBG$_O$ binding was severely affected only by mutations in the heel helix, while the binding of BG$_{ZP\text{-}C}$ was mostly unaffected. Fitting curves (red) were generated based on three consecutive repeats.

(**b** - **e**) Natural abundance methyl ^{1}H-^{13}C correlation NMR spectra indicating minimal perturbations in the chemical environment of binding affecting mutants of BG$_{ZP\text{-}C}$, indicating that the mutations lead to localized structural alterations rather than a widespread change in conformation.

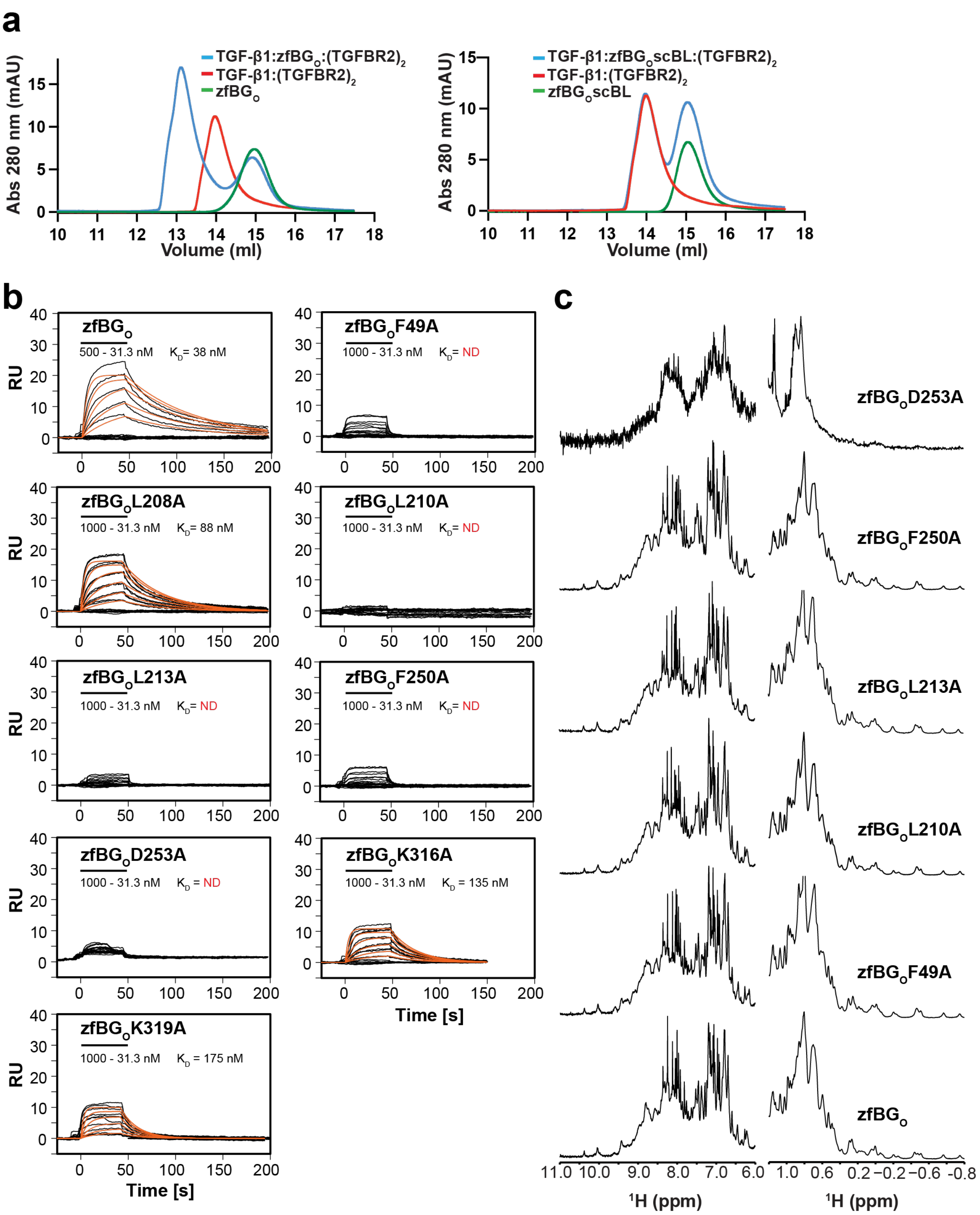
a
TGF-β1:zfBGo:(TGFBR2)2
TGF-β1:(TGFBR2)2
zfBGo
Abs 280 nm (mAU)
Volume (ml)
TGF-β1:zfBGoscBL:(TGFBR2)2
TGF-β1:(TGFBR2)2
zfBGoscBL
Abs 280 nm (mAU)
Volume (ml)
b
RU
zfBGo
500 - 31.3 nM KD= 38 nM
zfBGoF49A
1000 - 31.3 nM KD= ND
zfBGoL208A
1000 - 31.3 nM KD= 88 nM
zfBGoL210A
1000 - 31.3 nM KD= ND
zfBGoL213A
1000 - 31.3 nM KD= ND
zfBGoF250A
1000 - 31.3 nM KD= ND
zfBGoD253A
1000 - 31.3 nM KD = ND
zfBGoK316A
1000 - 31.3 nM KD = 135 nM
zfBGoK319A
1000 - 31.3 nM KD = 175 nM
Time [s]
Time [s]
c
zfBGoD253A
zfBGoF250A
zfBGoL213A
zfBGoL210A
zfBGoF49A
zfBGo
1H (ppm)
1H (ppm)

Supplementary Figure 6. zfBG$_O$ mutations within the TGF-β:zfBG$_O$ binding interface.

(**a**) SEC profiles of TGF-β1:zfBG$_O$:(TGFBR2)$_2$ and TGF-β1:(TGFBR2)$_2$ complexes (left panel) or TGF-β1:zfBG$_O$scBL:(TGFBR2)$_2$ and TGF-β1:(TGFBR2)$_2$ complexes (right panel), where zfBG$_O$scBL corresponds to zfBG$_O$ with BL fully replaced with unrelated sequence (scrambled). No shift in the TGF-β1:zfBG$_O$scBL:(TGFBR2)$_2$ elution volume together with the presence of second peak eluted at the BG$_O$ volume is indicative of zfBG$_O$scBL not forming a complex with TGF-β1:(TGFBR2)$_2$.

(**b**) SPR sensorgrams of zfBG$_O$ mutants injected in a 2-fold dilution series (black) show binding to immobilized TGF-β2. Fitting curves (red) were derived from data collected in two consecutive repeats.

(**c**) 1D proton NMR spectra, covering both the amide and methyl spectral regions. The spectra underscore that mutations predominantly result in localized alterations to the binding interface, without extensive perturbation of the overall protein structure. Notably, the D253A mutant exhibits signal broadening across the spectra, hinting at more widespread structural changes that may suggest alterations in protein folding.

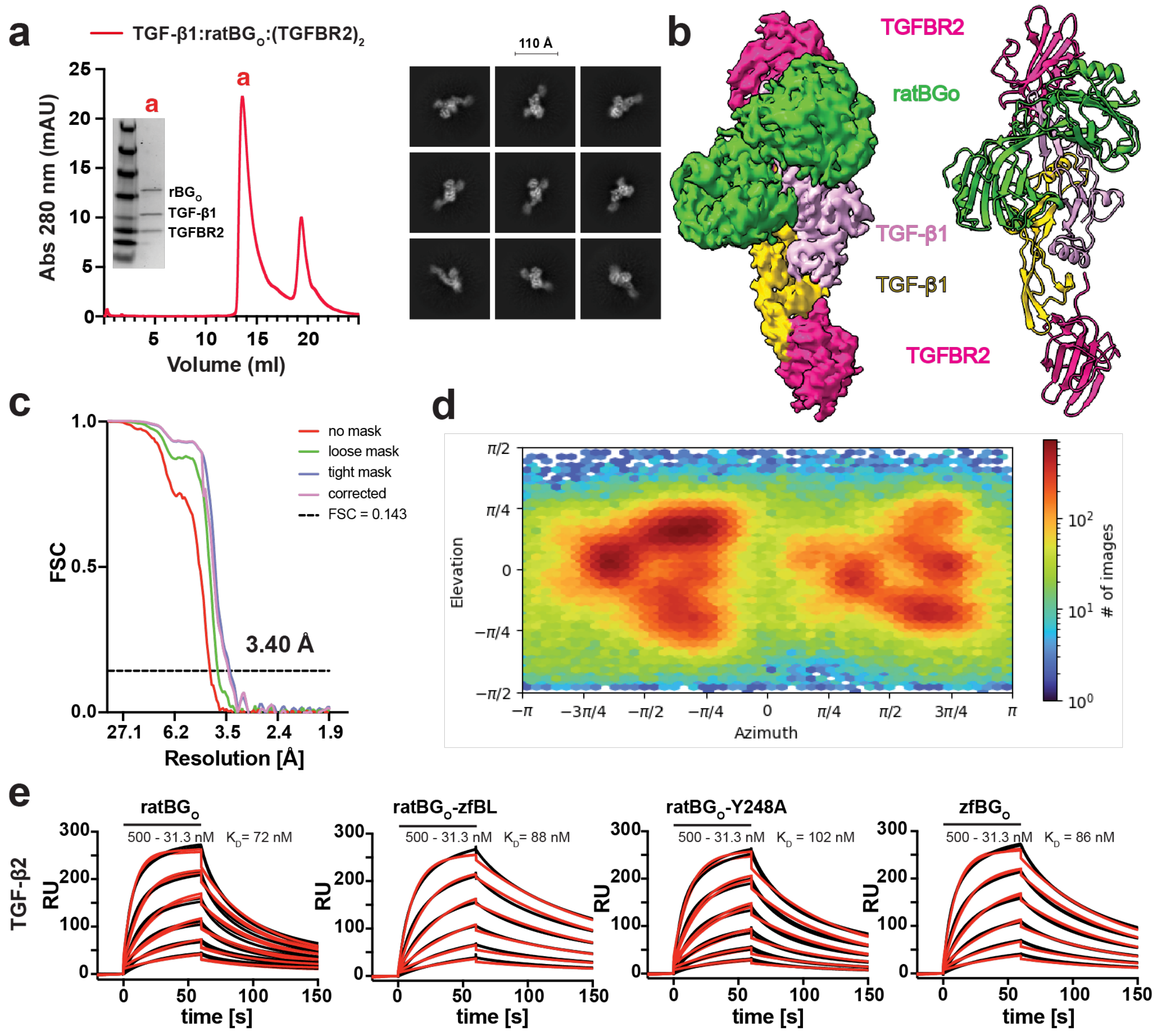

Supplementary Figure 7. Structural details of the TGF-β1:ratBG$_O$:(TGFBR2)$_2$ complex and SPR validation of the ratBG$_O$ mutants binding to TGF-β.

(**a**) SEC purification profile of TGF-β1:ratBG$_O$:(TGFBR2)$_2$ demonstrates distinct peak of assembled complex (red), as assessed by SDS-PAGE (inset). Selected 2D classes provide an overview of the dataset quality and coverage of different views. Scale bar, 110 Å.

(**b**) Cryo-EM map with a model built into the density (PDB: 9FDY).

(**c, d**) FSC curve of the final map refinement (**c**) with particle view distribution (**d**).

(**e**) SPR sensorgrams showing response to a 2-fold dilution series of injections (black) of ratBG$_O$, zfBG$_O$, as well as two mutants, ratBG$_O$zfBL and ratBG$_O$–Y248A, over immobilized TGF-β2 on the chip surface. Fitting curves (red) were derived from data collected in three consecutive repeats.

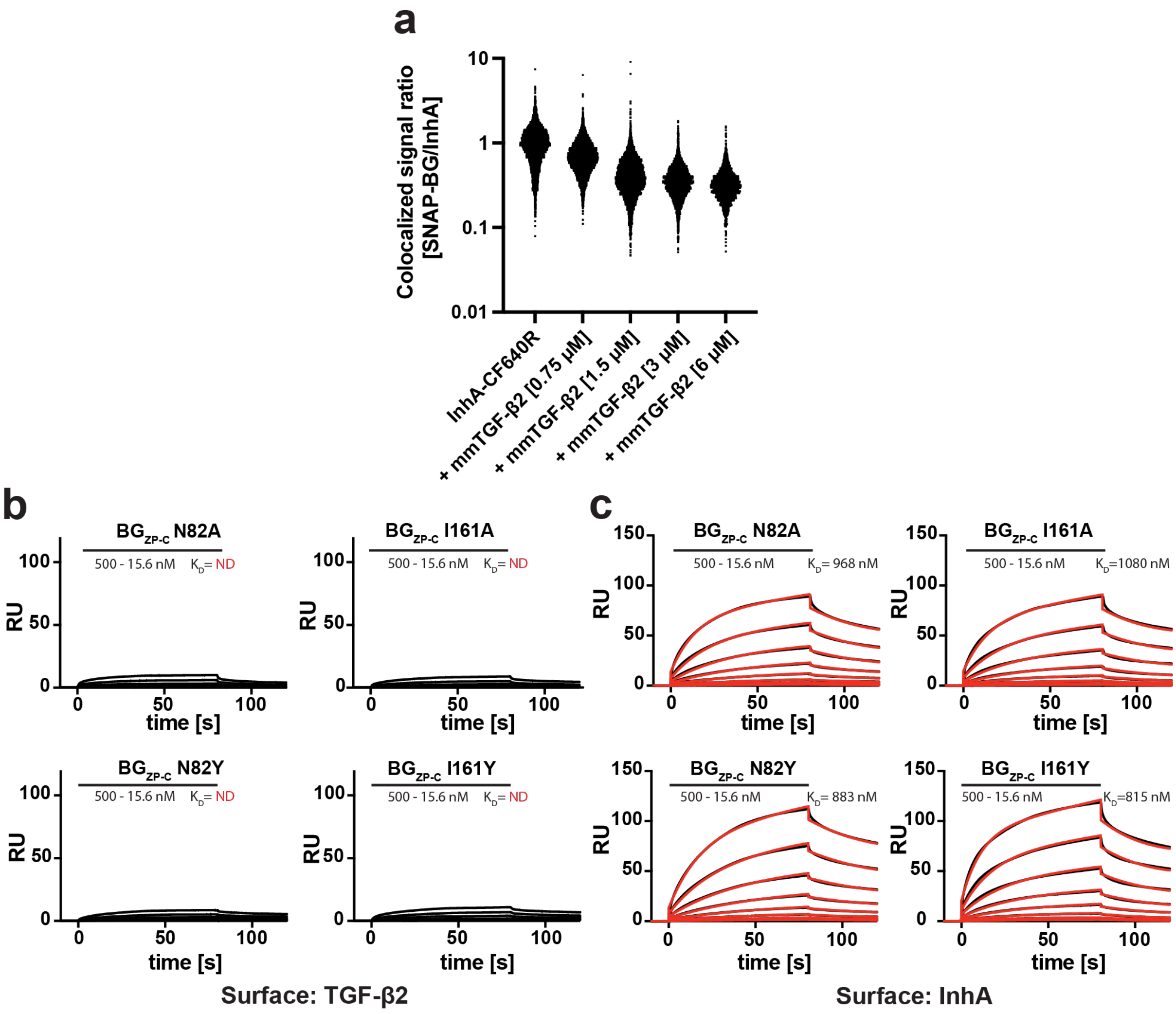

Supplementary Figure 8. InhA binds BG$_{ZP-C}$ using a different but overlapping interface to the TGF-βs.

(**a**) Quantification of the concentration-dependent effect of mmTGF-β2 on InhA-CF640R signal strength. Each data point represents the SNAP-BG signal to InhA signal ratio within a segmented region of the image. The analysis was performed on three images, each corresponding to an independent biological replicate (n=3).

(**b**, **c**) SPR sensorgrams showing response to 2-fold serial dilutions of selected mutants of BG$_{ZP-C}$ (black) injected over immobilized TGF-β2 (**b**) or InhA (**c**). Fitting curves (red) are derived from the data collected in three consecutive repeats.

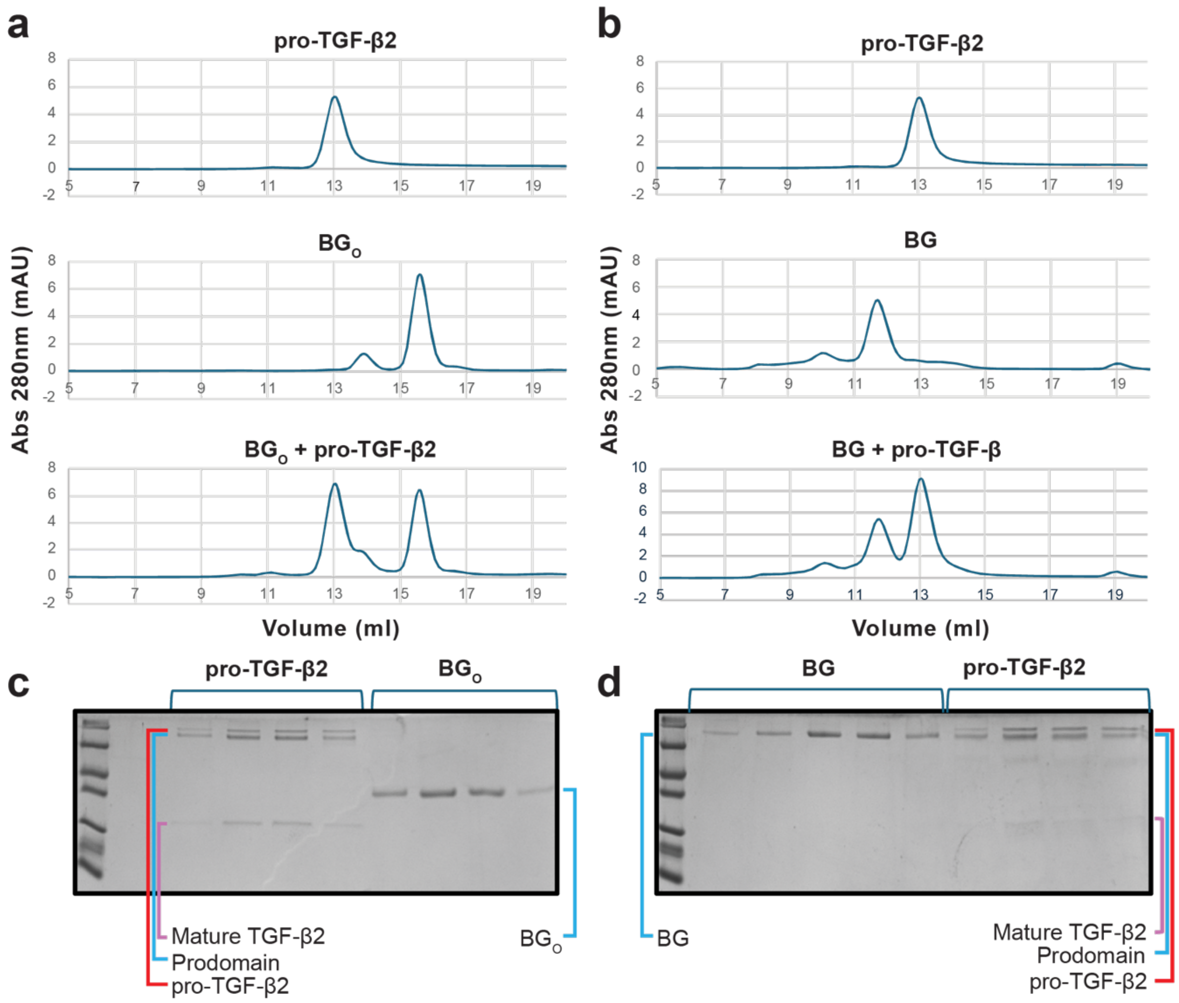

Supplementary Figure 9. BG or isolated BG_O do not form complexes with pro-TGF-β2.

(**a**, **c**) SEC profiles of the pro-TGF-β2, BG_O, or mixture of both incubated together for 30 min (**a**). Fractions containing proteins from the last profile were visualized by SDS-PAGE (**c**).

(**b**, **d**) SEC profiles of the pro-TGF-β2, BG, or mixture of both incubated together for 30 min (**b**). Fractions containing proteins from the last profile were visualized by SDS-PAGE (**d**).

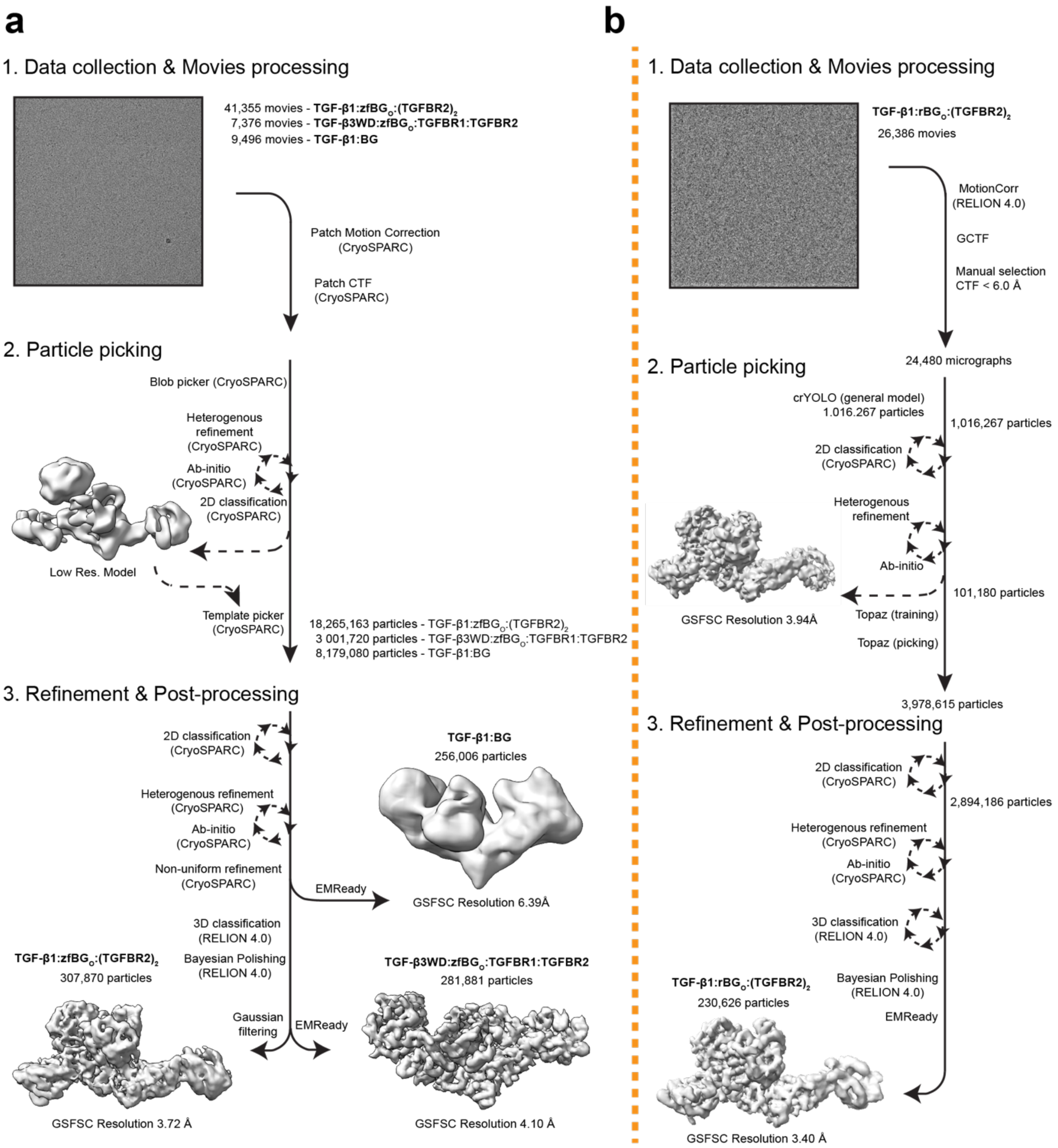

Supplementary Figure 10. Cryo-EM data processing workflow.

(**a**, **b**) A schematic representation of the Cryo-EM data processing pipeline of TGF-β1:zfBG$_O$:(TGFBR2)$_2$, TGF-β3WD:zfBG$_O$:TGFBR1:TGFBR2 and TGF-β1:BG (**a**), or TGF-β1:rBG$_O$:(TGFBR2)$_2$ (**b**) that was collected on grids covered with graphene oxide The workflow illustrates the steps from raw micrograph collection, particle picking, 2D classification, 3D reconstruction, refinement, post-processing and final map generation.

**Supplementary Table 1.** X-ray data collection and refinement parameters. Values in parentheses represent the highest resolution shell.		
**X-ray data collection**		
Specimen	mmTGF-β2:BG_{ZP-C}	TGF-β3WD:BG_O:TGFBR1:TGFBR2
Accession code	PDB: 8DC0	PDB: 9B9F
X-ray source	APS BEAMLINE 24-ID-E	APS BEAMLINE 24-ID-C
Space group	*I*222	$P2_12_12$
Cell Dimensions:		
a, *b*, *c* (Å)	76.5, 90.5, 98.9	195.1, 200.9, 47.4
α, *β*, *γ* (°)	90, 90, 90	90, 90, 90
Wavelength (Å)	0.97918	0.97918
Resolution range (Å)	66.74-1.93 (2.03-1.93)	139.98-3.0 (3.16-3.0)
Unique reflections	26,132 (3,763)	38,615 (5,562)
Multiplicity	13.0 (13.1)	13.4 (13.9)
Completeness (%)	100 (99.9)	100 (100)
Mean *I*/σ*I*	9.3 (1.0)	10.5 (0.6)
R_{pim}(I)	0.052 (0.967)	0.045 (1.43)
$CC_{1/2}$	0.99 (0.41)	0.99 (0.41)
**Refinement**		
Reflections used in refinement	26,107	38,508
Reflections used for R-free	1,998	2,011
R-work	0.219	0.235
R-free	0.257	0.278
Number of non-hydrogen atoms:		
Proteins	2,046	11,078
Water	85	0
Ramachandran favored (%)	96.39	93.34
Ramachandran allowed (%)	3.61	6.66
Ramachandran outliers (%)	0.00	0.00
Rotamer outliers (%)	1.28	1.09
Clashscore	7.88	9.29
Average B-factor	54.87	156.18

**Supplementary Table 2.** Cryo-EM data collection and refinement parameters				
Specimen	TGF-β3WD: zfBGO: TGFBR1: TGFBR2	TGF-β1: zfBGO: $(TGFBR2)_2$	TGF-β1:BG	TGF-β1: ratBGO: $(TGFBR2)_2$
Accession code	EMD-50519 PDB: 9FK5	EMD-50524 PDB: 9FKP	EMD-50326	EMD-50333 PDB: 9FDY
**Data collection**				
Magnification	168675 x	194444 x	168675 x	152009 x
Voltage	300	300	300	300
Electron exposure (e-/ Å2)	50	50	55	50
Defocus range (μm)	-0.75 to -2.25	-0.75 to -2.25	-0.75 to -2.25	-1.5 to -3.5
Pixel size	0.83	0.72	0.83	0.921
Symmetry imposed	C1	C1	C1	C1
Initial particle images	3 001 720	18 265 163	8 179 080	3 978 615
Final particle images	281 881	307 870	256 006	230 626
Map resolution (Å)	4.10	3.72	6.39	3.40
cFAR	0.05	0.01	0.36	0.4
SCF	0.89	0.42	0.97	0.80
FSC threshold	0.5	0.5	0.5	0.143
**Refinement**				
Map sharpening B factor (A2)	99.9	63.5	457	172.1
Chains	5	5	3	5
Non-hydrogen atoms	5766	6093	NA	6120
Protein residues	735	769	NA	770
Ligands	0	0	0	0
**Validation**				
- MolProbity score	2.03	1.86	NA	2.13
- Clashcore	7.29	7.06	NA	9.26
- Poor rotamers (%)	2.25%	1.86%	NA	2.56%
**Ramachandran plot**				
- Favored	94.76%	96.05%	NA	95.39%
- Allowed	5.24%	3.95%	NA	4.61%
- Disallowed	0.00%	0.00%	NA	0.00%

Supplementary Table 3: Key resources		
REAGENT	SOURCE	IDENTIFIER
**Antibodies**		
Anti-phospho-Smad2 (IF, Dilution: 1 in 500)	Cell Signaling Technology	Cat# 3108; RRID: AB_490941
Anti-SNAP-tag (Polyclonal, Dilution 1:1000)	Invitrogen	Cat# CAB4255<br>RRID: AB_10710011
Anti-Betaglycan (Polyclonal, Dilution 1:1000)	R&D	Cat#AF5034<br>RRID: AB_2202608
HRP-conjugated anti-rabbit secondary antibodies (IF, Dilution: 1:5000)	Dako	Cat# P0448<br>RRID: AB_2617138
HRP-conjugated anti-goat secondary antibodies (IF, Dilution: 1:5000)	Dako	Cat# P0449, RRID:AB_2617143
**Bacterial strains**		
E. coli BL21(DE3)	EMD-Milipore	Cat# 69450-3
**Chemicals, peptides, and recombinant proteins**		
Alexa Fluor™ 647 NHS ester	ThermoFisher Scientific	Cat# A20006
CF®640R Succinimide ester	Biotium	Cat# 92108
CF®568 Succinimide ester	Biotium	Cat# 92131
BG-NH_2 O6-(4-Aminomethyl-benzyl)guanine	AAT Bioquest	Cat# 1256-AAT
DAPI	Sigma	Cat# 10236276001
Recombinant BMP2	R&D systems	Cat# 355-BM-100/CF
Recombinant BMP4	R&D systems	Cat# 314-BP-050/CF
Recombinant Activin A	R&D systems	Cat# 338-AC-050/CF
Recombinant Inhibin A	R&D systems	Cat# 8506-AB-010/CF
Recombinant TGF-β1	This study	

Recombinant TGF-β2	This study	
Recombinant TGF-β3	This study	
Recombinant TGF-β3WD	This study	
Recombinant TGFBR2	This study	
Recombinant TGFBR1	This study	
Recombinant BG	This study	
Recombinant ratBG$_{ZP\text{-}C}$ (wilde type + all mutants)	This study	
Recombinant ratBG$_{O}$ (wilde type + all mutants)	This study	
Recombinant zfBG$_{O}$ (wilde type + all mutants)	This study	
Paraformaldehyde solution 4% in PBS	ThermoFisher Scientific	Cat# J19943-K2
ProLong™ antifade reagent	Invitrogen	Cat# P36930
EDC	Pierce	Cat# PG82079
EZ-Link Amine-PEG3-Biotin	ThermoFisher Scientific	Cat# 21347
Sulfo-NHS (N-hydroxysulfosuccinimide)	Pierce	Cat# PG82071
DMSO	Sigma	Cat# D1435
Amine Coupling Kit	Cytiva	Cat# BR100050
IPTG	Goldbio	Cat# I2481C
Poly-L-Lysine	Cultrex	Cat# 3438-100-01
FuGene® HD	Promega	Cat# E2311
ExpiFectamine™ 293 Transfection Kit	Gibco	Cat# A14525
Lipofectamine 3000	ThermoFisher Scientific	Cat# L3000015
Fetal Bovine Serum	ThermoFisher Scientific	Cat# 10270-106

DMEM	ThermoFisher Scientific	Cat# 41966029
FreeStyle™ 293 Expression Medium	Gibco	Cat# 12338-018
Penicillin-Streptomycin	ThermoFisher Scientific	Cat# 15140122
NeXtal PEGs II Suite	Molecular Dimensions	Cat#130916
Graphene Oxide	Sigma	Cat# 763705
MES	Sigma	Cat# M5287
HEPES	Sigma	Cat# H3375
Tris-HCl	ThermoFisher Scientific	Cat# 15506017
Sodium acetate	Sigma	Cat# S8750
NaCl	Sigma	Cat# S7653
Glutathione reduced	Sigma	Cat# G6013
Glutathione oxidized	Sigma	Cat# G4376
CHAPS	Goldbio	Cat# C-080-1
DTT	Sigma	Cat# D9779
Acetic acid, glacial	Fisher Scientific	Cat# 10304980
Isopropanol	Fisher Scientific	Cat# 10315720
Acetonitrile	Fisher Scientific	Cat# 10407440
**Deposited Data**		
X-ray structure of complex: mmTGF-β2:BG_{ZP-C}	PDB, this study	8DC0
X-ray structure of complex: TGF-β3WD: BG_O:TGFBR1:TGFBR2	PDB, this study	9B9F
Cryo-EM map of complex: TGF-β3WD: BG_O:TGFBR1:TGFBR2	PDB, this study	9FK5, EMD-50519

Cryo-EM map of complex: TGF–β1:BG_O:$(TGFBR2)_2$	PDB, this study	9FKP, EMD-50326
Cryo-EM map of complex: TGF–β1: ratBG_O:$(TGFBR2)_2$	PDB, this study	9FDY, EMD-50333
Cryo-EM map of complex: TGF–β1:BG	PDB, this study	EMD-50326
**Experimental models: Cell Lines**		
HEK293T	Francis Crick Institute Cell Services	N/A
Expi293F	Invitrogen	Cat# A14527
L6E9	Ref [1]	N/A
HEK293T SNAP-BG	This study	N/A
**Recombinant DNA**		
pCDNA3.1+-SNAP-BG	This study	N/A
pCDNA3.1+-BG	Ref [2]	N/A
pCDNA3.1+-zfBG_O	Ref [3]	N/A
pCDNA3.1+-ratBG_O	Ref [2]	N/A
pCDNA3.1+-ratBG_O-zfBL	This study	N/A
pCDNA3.1+-ratBG_O-Y248A	This study	N/A
pCDNA3.1+-zfBG_O-scBL	This study	N/A
pCDNA3.1+-ratBG_O-F49A	This study	N/A
pCDNA3.1+-ratBG_O-L208A	This study	N/A
pCDNA3.1+-ratBG_O-L210A	This study	N/A
pCDNA3.1+-ratBG_O-L213A	This study	N/A
pCDNA3.1+-ratBG_O-F250A	This study	N/A
pCDNA3.1+-ratBG_O-D253A	This study	N/A
pCDNA3.1+-ratBG_O-K316A	This study	N/A

pCDNA3.1+-ratBG$_O$-K319A	This study	N/A
pCDNA3.1+-ratBG$_O$-zfBL	This study	N/A
pCDNA3.1+-ALB-BG$_{ZP-C}$	Ref [4]	N/A
pCDNA3.1+-ALB-BG$_{ZP-C}$-N82A	This study	N/A
pCDNA3.1+-ALB-BG$_{ZP-C}$-N82Y	This study	N/A
pCDNA3.1+-ALB-BG$_{ZP-C}$-K86A	This study	N/A
pCDNA3.1+-ALB-BG$_{ZP-C}$-D88A	This study	N/A
pCDNA3.1+-ALB-BG$_{ZP-C}$-E130A	This study	N/A
pCDNA3.1+-ALB-BG$_{ZP-C}$-L143A	This study	N/A
pCDNA3.1+-ALB-BG$_{ZP-C}$-S155A	This study	N/A
pCDNA3.1+-ALB-BG$_{ZP-C}$-A158A	This study	N/A
pCDNA3.1+-ALB-BG$_{ZP-C}$-A158E	This study	N/A
pCDNA3.1+-ALB-BG$_{ZP-C}$-I161A	This study	N/A
pCDNA3.1+-ALB-BG$_{ZP-C}$-I161Y	This study	N/A
pCDNA3.1+-ALB-BG$_{ZP-C}$-W162A	This study	N/A
pCDNA3.1+-ALB-BG$_{ZP-C}$-M164A	This study	N/A
pCDNA3.1+-ALB-BG$_{ZP-C}$-W162A/M164A	This study	N/A
pCDNA3.1+-ALB-BG$_{ZP-C}$-Q166A	This study	N/A
pCDNA3.1+-proTGF-β1	Ref [5]	N/A
pCDNA3.1+-proTGF-β2	This study	N/A
pCDNA3.1+-proTGF-β2-(E99K, L101Y, S102Q, N103D)	This study	N/A
pCDNA3.1+-proTGF-β2-(E99K, S102Q, N103D)	This study	N/A
pCDNA3.1+-proTGF-β2-(E99K, S102Q)	This study	N/A
pCDNA3.1+-proTGF-β2-(S102Q, N103D)	This study	N/A
pET32a-TGF-β2	Ref [4]	N/A
pET32a-TGF-β2-(Q57A, K60A, L64A)	This study	N/A
pET32a-TGF-β2-(Y50A, L51A)	This study	N/A
pET32a-TGF-β3	Ref [6]	N/A

pET32a-TGF-β3D	Ref [5]	N/A
pET32a-mmTGF-β27M	Ref [7]	N/A
pET32a-BG$_{ZP-C}$	Ref [2]	N/A
pET32a-TGFBR1(31-115)	Ref [6]	N/A
pET32a-TGFBR2(42-153)	Ref [6]	N/A
pCMV5-BG$_{ZP-C}$	Ref [1]	N/A
pCMV5-BG$_{ZP-C}$-(N82A)	This study	N/A
pCMV5-BG$_{ZP-C}$-(I161A)	This study	N/A
pCMV5-BG$_{ZP-C}$-(N82A, I161A)	This study	N/A
CAGA$_{12}$-luciferase	Ref [8]	N/A
**Software**		
FIJI (ImageJ)	Ref [9]	https://imagej.net/Fiji/Downloads
Prism 10	Graph Pad	https://www.graphpad.com/scientific-software/prism/
TopSpin 4.0	Bruker Biospin	https://www.bruker.com/en/products-and-solutions/mr/nmr-software/topspin.html
NMRPipe	Ref [10]	https://www.ibbr.umd.edu/nmrpipe/install.html
CCPNMR	Ref [11]	https://ccpn.ac.uk/software/downloads/
CCP4 8.0	Ref [12]	https://www.ccp4.ac.uk/download/
Phenix 1.21	Ref [13]	https://phenix-online.org/download/
autoPROC	Ref [14]	https://www.globalphasing.com/autoproc/
PHASER	Ref [15]	https://www.phaser.cimr.cam.ac.uk/index.php/Phaser_Crystallographic_Software

Pointless	Refs [16,17]	https://www.ccp4.ac.uk/html/pointless.html
Aimless	Refs [16,17]	https://www.ccp4.ac.uk/html/aimless.html
Ctruncate	Refs [16,17]	https://www.ccp4.ac.uk/html/ctruncate.html
XDS	Ref [18]	https://wiki.uni-konstanz.de/xds/index.php/Main_Page
Coot	Ref [19]	https://www2.mrc-lmb.cam.ac.uk/personal/pemsley/coot/
ChimeraX1.6	Ref [20]	https://www.cgl.ucsf.edu/chimerax/
CrYOLO	Ref [21]	https://cryolo.readthedocs.io/en/stable/
Relion 4.0	Ref [22]	https://relion.readthedocs.io/en/release-4.0/
CryoSparc	Ref [23]	https://cryosparc.com
ISOLDE	Ref [24]	https://tristanic.github.io/isolde/
MotionCor2 1.5.0	Ref [25]	https://emcore.ucsf.edu/ucsf-software
GCTF 1.18	Ref [26]	https://sbgrid.org/software/titles/gctf
Namdinator	Ref [27]	https://namdinator.au.dk/namdinator/
AlphaFold2	Ref [28]	https://github.com/google-deepmind/alphafold
TOPAZ	Ref [29]	https://emgweb.nysbc.org/topaz.html
PDBePISA	Ref [30]	https://www.ebi.ac.uk/pdbe/pisa/
Jalview	Ref [31]	https://www.jalview.org/

Clustal Omega	Ref [32]	http://www.clustal.org/
Scrubber	Biologic Software	http://www.biologic.com.au/scrubber.html
BiaEval	Biacore	https://www.cytivalifesciences.com/en/us/support/software/biacore-downloads
**Other**		
Series S CM-4 SPR sensor chip	Cytiva	Cat# BR100534
Classic CM-5 SPR sensor chip	Cytiva	Cat# BR100399
Superdex® 200 10/300 GL	Cytiva	Cat# GE17-5175-01
HiLoad® 16/600 Superdex® 200 pg	Cytiva	Cat# GE28-9893-35
Ni-NTA agarose	Qiagen	Cat# 30210
cOmpleteTM His-Tag Purification column	Roche	Cat# 06781535001
Mono S® 5/50 GL	Sigma	Cat# GE17-5168-01
SP Sepharose	Cytiva	Cat# GE17-0729-10
UltrAuFoil R1.2/1.3	Quantifoil	Cat# TEM-Q350AR13A

Supplementary References

1. Esparza-Lopez, J. et al. Ligand binding and functional properties of betaglycan, a co-receptor of the transforming growth factor-β superfamily. Specialized binding regions for transforming growth factor-β and inhibin A. *J Biol Chem* **276**, 14588-96 (2001).
2. Villarreal, M.M. et al. Binding Properties of the Transforming Growth Factor-β Coreceptor Betaglycan: Proposed Mechanism for Potentiation of Receptor Complex Assembly and Signaling. *Biochemistry* **55**, 6880-6896 (2016).
3. Kim, S.K. et al. Structural Adaptation in Its Orphan Domain Engenders Betaglycan with an Alternate Mode of Growth Factor Binding Relative to Endoglin. *Structure* **27**, 1427-1442 e4 (2019).

4. Henen, M.A. et al. TGF-β2 uses the concave surface of its extended finger region to bind betaglycan's ZP domain via three residues specific to TGF-β and inhibin-α. *J Biol Chem* **294**, 3065-3080 (2019).
5. Huang, T., Schor, S.L. & Hinck, A.P. Biological activity differences between TGF-β1 and TGF-β3 correlate with differences in the rigidity and arrangement of their component monomers. *Biochemistry* **53**, 5737-49 (2014).
6. Groppe, J. et al. Cooperative assembly of TGF-β superfamily signaling complexes is mediated by two disparate mechanisms and distinct modes of receptor binding. *Mol Cell* **29**, 157-68 (2008).
7. Kim, S.K. et al. An engineered transforming growth factor β (TGF-β) monomer that functions as a dominant negative to block TGF-β signaling. *J Biol Chem* **292**, 7173-7188 (2017).
8. Dennler, S. et al. Direct binding of Smad3 and Smad4 to critical TGF β-inducible elements in the promoter of human plasminogen activator inhibitor-type 1 gene. *EMBO J* **17**, 3091-100 (1998).
9. Schindelin, J. et al. Fiji: an open-source platform for biological-image analysis. *Nat Methods* **9**, 676-82 (2012).
10. Delaglio, F. et al. NMRPipe: a multidimensional spectral processing system based on UNIX pipes. *J Biomol NMR* **6**, 277-93 (1995).
11. Vranken, W.F. et al. The CCPN data model for NMR spectroscopy: development of a software pipeline. *Proteins* **59**, 687-96 (2005).
12. Agirre, J. et al. The CCP4 suite: integrative software for macromolecular crystallography. *Acta Crystallogr D Struct Biol* **79**, 449-461 (2023).
13. Adams, P.D. et al. PHENIX: a comprehensive Python-based system for macromolecular structure solution. *Acta Crystallogr D Biol Crystallogr* **66**, 213-21 (2010).
14. Vonrhein, C. et al. Data processing and analysis with the autoPROC toolbox. *Acta Crystallogr D Biol Crystallogr* **67**, 293-302 (2011).
15. McCoy, A.J. et al. Phaser crystallographic software. *J Appl Crystallogr* **40**, 658-674 (2007).
16. Evans, P. Scaling and assessment of data quality. *Acta Crystallogr D Biol Crystallogr* **62**, 72-82 (2006).
17. Evans, P.R. An introduction to data reduction: space-group determination, scaling and intensity statistics. *Acta Crystallogr D Biol Crystallogr* **67**, 282-92 (2011).
18. Kabsch, W. Xds. *Acta Crystallogr D Biol Crystallogr* **66**, 125-32 (2010).
19. Emsley, P. & Cowtan, K. Coot: model-building tools for molecular graphics. *Acta Crystallogr D Biol Crystallogr* **60**, 2126-32 (2004).
20. Meng, E.C. et al. UCSF ChimeraX: Tools for structure building and analysis. *Protein Sci* **32**, e4792 (2023).
21. Wagner, T. et al. SPHIRE-crYOLO is a fast and accurate fully automated particle picker for cryo-EM. *Commun Biol* **2**, 218 (2019).
22. Kimanius, D., Dong, L., Sharov, G., Nakane, T. & Scheres, S.H.W. New tools for automated cryo-EM single-particle analysis in RELION-4.0. *Biochem J* **478**, 4169-4185 (2021).
23. Punjani, A., Rubinstein, J.L., Fleet, D.J. & Brubaker, M.A. cryoSPARC: algorithms for rapid unsupervised cryo-EM structure determination. *Nat Methods* **14**, 290-296 (2017).

24. Croll, T.I. ISOLDE: a physically realistic environment for model building into low-resolution electron-density maps. *Acta Crystallogr D Struct Biol* **74**, 519-530 (2018).
25. Zheng, S.Q. et al. MotionCor2: anisotropic correction of beam-induced motion for improved cryo-electron microscopy. *Nat Methods* **14**, 331-332 (2017).
26. Zhang, K. Gctf: Real-time CTF determination and correction. *J Struct Biol* **193**, 1-12 (2016).
27. Kidmose, R.T. et al. Namdinator - automatic molecular dynamics flexible fitting of structural models into cryo-EM and crystallography experimental maps. *IUCrJ* **6**, 526-531 (2019).
28. Jumper, J. et al. Highly accurate protein structure prediction with AlphaFold. *Nature* **596**, 583-589 (2021).
29. Bepler, T. et al. Positive-unlabeled convolutional neural networks for particle picking in cryo-electron micrographs. *Nat Methods* **16**, 1153-1160 (2019).
30. Krissinel, E. & Henrick, K. Inference of macromolecular assemblies from crystalline state. *J Mol Biol* **372**, 774-97 (2007).
31. Waterhouse, A.M., Procter, J.B., Martin, D.M., Clamp, M. & Barton, G.J. Jalview Version 2--a multiple sequence alignment editor and analysis workbench. *Bioinformatics* **25**, 1189-91 (2009).
32. Sievers, F. et al. Fast, scalable generation of high-quality protein multiple sequence alignments using Clustal Omega. *Mol Syst Biol* **7**, 539 (2011).
