# Supplementary material for: Structures of TGF-β with betaglycan and signaling receptors reveal mechanisms of complex assembly and signaling
Source: Nat Commun. 2025 Feb 26;16:1778. doi: 10.1038/s41467-025-56796-9 (PMC11865472; doi:10.1038/s41467-025-56796-9)
Supplement: Supplementary file 2 — Description of Additional Supplementary Files [file 41467_2025_56796_MOESM2_ESM.pdf]

Description of Additional Supplementary Files

File name: Supplementary Data 1
Description: Alphafold model of BG_{ZP-C} in complex with TGF-β2.

File name: Supplementary Data 2
Description: Alphafold model of BG_{O} in complex with TGF-β3 and signaling receptors TGFBR1 and TGFBR2.
