# Supplementary material for: Structures of TGF-β with betaglycan and signaling receptors reveal mechanisms of complex assembly and signaling
Source: Nat Commun. 2025 Feb 26;16:1778. doi: 10.1038/s41467-025-56796-9 (PMC11865472; doi:10.1038/s41467-025-56796-9)
Supplement: Supplementary file 5 — Reporting Summary [file 41467_2025_56796_MOESM5_ESM.pdf]

nature portfolio

Corresponding author(s): Caroline S Hill and Andrew P Hinck

Last updated by author(s): Jan 7, 2025

Reporting Summary

Nature Portfolio wishes to improve the reproducibility of the work that we publish. This form provides structure for consistency and transparency in reporting. For further information on Nature Portfolio policies, see our Editorial Policies and the Editorial Policy Checklist.

Statistics

For all statistical analyses, confirm that the following items are present in the figure legend, table legend, main text, or Methods section.

n/a	Confirmed	
☐	☒	The exact sample size (*n*) for each experimental group/condition, given as a discrete number and unit of measurement
☒	☐	A statement on whether measurements were taken from distinct samples or whether the same sample was measured repeatedly
☐	☒	The statistical test(s) used AND whether they are one- or two-sided<br>*Only common tests should be described solely by name; describe more complex techniques in the Methods section.*
☒	☐	A description of all covariates tested
☒	☐	A description of any assumptions or corrections, such as tests of normality and adjustment for multiple comparisons
☐	☒	A full description of the statistical parameters including central tendency (e.g. means) or other basic estimates (e.g. regression coefficient) AND variation (e.g. standard deviation) or associated estimates of uncertainty (e.g. confidence intervals)
☐	☒	For null hypothesis testing, the test statistic (e.g. *F*, *t*, *r*) with confidence intervals, effect sizes, degrees of freedom and *P* value noted<br>*Give P values as exact values whenever suitable.*
☒	☐	For Bayesian analysis, information on the choice of priors and Markov chain Monte Carlo settings
☒	☐	For hierarchical and complex designs, identification of the appropriate level for tests and full reporting of outcomes
☒	☐	Estimates of effect sizes (e.g. Cohen's *d*, Pearson's *r*), indicating how they were calculated

Our web collection on statistics for biologists contains articles on many of the points above.

Software and code

Policy information about availability of computer code

Data collection	Diffraction data were collected at the Advanced Photon Source, Argonne, IL, NE-CAT beamlines. NMR data were collected using TopSpin 4.0, Both Krios microscopes at University of Pittsburgh and at Diamond were operated by EPU automated software, SPR data was collected suing Biacore Control Software (BCS) and confocal images were recorded using Leica Application Suite X (LAS X)
Data analysis	BiaEval, Scrubber, NMRPipe, CCPNMR, Phenix 1.21, autoPROC, PHASER, Pointless, Aimless, Ctruncate, XDS, coot, CrYOLO, Relion 4.0, MotionCor2 1.5.0, GCTF 1.18, Namidnator, Alphafold2-multimer, Fiji, Prism 10, ChimeraX 1.6, CryoSPARC, TOPAZ. The details of data processing and analysis are described in the materials & methods section.

For manuscripts utilizing custom algorithms or software that are central to the research but not yet described in published literature, software must be made available to editors and reviewers. We strongly encourage code deposition in a community repository (e.g. GitHub). See the Nature Portfolio guidelines for submitting code & software for further information.

Data

Policy information about availability of data

All manuscripts must include a data availability statement. This statement should provide the following information, where applicable:

- Accession codes, unique identifiers, or web links for publicly available datasets
- A description of any restrictions on data availability
- For clinical datasets or third party data, please ensure that the statement adheres to our policy

All coordinates, structure factors and CryoEM maps have been deposited in the Protein Data Bank (PDB) with the following accession numbers: 8DC0 (crystal structure of BGZP-C : mmTGF-β2 complex), 9B9F (Crystal structure of TGF-β3WD:BGO:TGFBR1:TGFBR2 complex), 9FK5 (CryoEM structure of TGF-β3WD:zfBGO:TGFBR1:TGFBR2 complex), 9FKP (CryoEM structure of TGF-β1:zfBGO:(TGFBR2)2 complex), and 9FDY (CryoEM structure of TGF-β1:ratBGO:(TGFBR2)2 complex). Additionally, CryoEM maps were deposited int the Electron Microscopy Data Bank (EMDB) with the following accession numbers: EMD-50519 (CryoEM structure of TGF-β3WD:zfBGO:TGFBR1:TGFBR2 complex), EMD-50524 (CryoEM structure of TGF-β1:zfBGO:(TGFBR2)2 complex), EMD-50333 (CryoEM structure of TGF-β1:ratBGO:(TGFBR2)2 complex) and EMD-50326 (CryoEM map of TGF-β1:BG complex). Source data are provided with this paper. Plasmids generated in this study are maintained in the laboratories of Andrew Hinck (ahinck@pitt.edu) and Caroline Hill (caroline.hill@crick.ac.uk) and will be made available upon request.

Research involving human participants, their data, or biological material

Policy information about studies with human participants or human data. See also policy information about sex, gender (identity/presentation), and sexual orientation and race, ethnicity and racism.

Reporting on sex and gender	N/A
Reporting on race, ethnicity, or other socially relevant groupings	N/A
Population characteristics	N/A
Recruitment	N/A
Ethics oversight	N/A

Note that full information on the approval of the study protocol must also be provided in the manuscript.

Field-specific reporting

Please select the one below that is the best fit for your research. If you are not sure, read the appropriate sections before making your selection.

☒ Life sciences ☐ Behavioural & social sciences ☐ Ecological, evolutionary & environmental sciences

For a reference copy of the document with all sections, see nature.com/documents/nr-reporting-summary-flat.pdf

Life sciences study design

All studies must disclose on these points even when the disclosure is negative.

Sample size	Sample size is not applicable as the paper contains no animal or human data.
Data exclusions	No data were excluded from the analyses
Replication	Biochemical and biophysical experiments were all repeated at least three times as stated.
Randomization	N/A
Blinding	N/A

Reporting for specific materials, systems and methods

We require information from authors about some types of materials, experimental systems and methods used in many studies. Here, indicate whether each material, system or method listed is relevant to your study. If you are not sure if a list item applies to your research, read the appropriate section before selecting a response.

Materials & experimental systems

n/a	Involved in the study
☐	☒ Antibodies
☐	☒ Eukaryotic cell lines
☒	☐ Palaeontology and archaeology
☒	☐ Animals and other organisms
☒	☐ Clinical data
☒	☐ Dual use research of concern
☒	☐ Plants

Methods

n/a	Involved in the study
☒	☐ ChIP-seq
☒	☐ Flow cytometry
☒	☐ MRI-based neuroimaging

Antibodies

Antibodies used

Anti-phospho-Smad2 (Western blot, Dilution: 1 in 500, Cell Signaling Technology, Cat#3108 RRID AB_490941), Anti-SNAP-tag (Western blot, Dilution 1:1000, Invitrogen, Cat# CAB4255, RRID AB_10710011), Anti-Betaglycan (Western blot, Dilution 1:1000, R&D, Cat# AF5034 RRID: AB_2202608), HRP-conjugated anti-rabbit secondary antibodies (Western blot, Dilution: 1:5000, Dako, Cat# P0448, RRID: AB_2617138), HRP-conjugated anti-goat secondary antibodies (Western blot, Dilution: 1:5000, Dako Cat# P0449, RRID:AB_2617143).

Validation

Anti-phospho-Smad2 (Cat# 3108) Cell Signaling Technology: https://www.cellsignal.co.uk/products/primary-antibodies/phospho-smad2-ser465-467-138d4-rabbit-mab/3108

Antibody has been successfully used multiple times in the Hill lab:
Gori et al. Elife 10 (2021): e63545.
Ramachandran et al. Elife 7 (2018): e31756.
Miller et al. Cell Reports 25.7 (2018): 1841-1855.
Guglielmi et al. Nature Communications 12.1 (2021): 6374.

Anti-SNAP-tag (Invitrogen, Cat# CAB4255)
https://www.thermofisher.com/antibody/product/SNAP-Tag-Antibody-Polyclonal/CAB4255

Antibody was successfully used multiple times:
Kim et al. PLoS biology 16.12 (2018): e2006660.
Seshire et al. Leukemia 26.6 (2012): 1338-1347.
Dunleavy et al. Nucleus 2.2 (2011): 146-157.
Winfield et al. Frontiers in Endocrinology 12 (2022): 792912.

Anti-Betaglycan (R&D, Cat# AF5034)
https://www.bio-techne.com/p/antibodies/mouse-tgf-beta-riii-antibody_af5034#tab-citations_reviews

The antibody has been used successfully multiple times
Becker et al Frontiers in Immunology 9 (2018): 688.
Alghetaa et al J Cell Mol Med 22.5 (2018): 2644-2655
Becker et al U.S. Patent No. 11,478,531. 25 Oct. 2022

HRP-conjugated anti-rabbit secondary antibodies (Dako, Cat# P0448)
https://www.agilent.com/en/product/specific-proteins/elisa-kits-accessories/goat-anti-rabbit-immunoglobulins-hrp-affinity-isolated-2717113?srsltid=AfmBOoqOe8dGE7I4abYJmC-ij0iYr4wBaAOzbG637r9X6Z7OIFoMqDRl

Antibody has been cited 1943 times
https://www.citeab.com/antibodies/3288347-p0448-goat-anti-rabbit-immunoglobulins-hrp-affinity

HRP-conjugated anti-goat secondary antibodies (Dako, Cat# P0449)
https://www.agilent.com/store/productDetail.jsp?catalogId=P044901-2

Antibody has been cited 447 times
https://www.citeab.com/antibodies/3288331-p0449-rabbit-anti-goat-immunoglobulins-hrp-affinity

Eukaryotic cell lines

Policy information about cell lines and Sex and Gender in Research

Cell line source(s)

HEK293T cells were obtained from the Francis Crick Institute Cell Services.
L6E9 cells were obtained from Instituto de Fisiología Celular, Universidad Nacional Autónoma de México.
Expi293F cells were obtained from Invitrogen.

Authentication

HEK293T cells were authenticated using short tandem repeat (STR) profiling.
L6E9 cells' response to ligands and the phenotype were consistent with published literature.

	Expi293F cells which were used for protein expression, behaved functionally as expected consistent with the published literature.
Mycoplasma contamination	All cell lines were certified mycoplasma negative by the Francis Crick Institute Cell Services and for the L6E9 line, also at McGill University.
Commonly misidentified lines (See ICLAC register)	No commonly misidentified lines were used

Plants

Seed stocks	N/A
Novel plant genotypes	N/A
Authentication	N/A
